# Nasal Continuous Positive Airway Pressure vs. Nasal Intermittent Positive Pressure Ventilation as Initial Treatment After Birth in Extremely Preterm Infants

**DOI:** 10.3389/fped.2022.870125

**Published:** 2022-04-25

**Authors:** Yasemin Ezgi Kostekci, Emel Okulu, Batuhan Bakirarar, Elvis Kraja, Omer Erdeve, Begum Atasay, Saadet Arsan

**Affiliations:** ^1^Division of Neonatology, Department of Pediatrics, Faculty of Medicine, Ankara University, Ankara, Turkey; ^2^Department of Biostatistics, Faculty of Medicine, Ankara University, Ankara, Turkey

**Keywords:** non-invasive respiratory support, nasal continuous positive pressure ventilation, nasal intermittent positive pressure ventilation, extremely preterm, respiratory distress syndrome

## Abstract

**Objective:**

Non-invasive respiratory support strategies are known to reduce the complications of invasive mechanical ventilation in preterm infants. Nasal continuous positive airway pressure (NCPAP) and nasal intermittent positive pressure ventilation (NIPPV) are commonly used ones. The recent meta-analyses indicated that early NIPPV did appear to be superior to NCPAP for decreasing respiratory failure and the need for intubation among preterm infants with respiratory distress syndrome (RDS). The aim of the study was to compare the short-term outcomes of extremely preterm infants who received NCPAP or NIPPV as an initial treatment of RDS.

**Methods:**

This retrospective study included infants born before 29 weeks' gestation between 1 January 2018 and 31 December 2021 who received non-invasive respiratory support with NCPAP or NIPPV. For every infant included in the cohort, only the first episode of NCPAP or NIPPV as initial treatment was evaluated. The primary outcome was the need for intubation within 72 h, and the secondary outcomes were the need for intubation within 7 days, administration of surfactant, prematurity-related morbidities, mortality, and death or bronchopulmonary dysplasia (BPD).

**Results:**

During the study period, there were 116 inborn admissions of preterm infants born <29 weeks' gestation and 60 of them met the inclusion criteria. Of these, 31 (52%) infants received NCPAP while 29 (48%) infants received NIPPV at the first hours after birth. There were no differences in the baseline demographics between the groups (*p* > 0.05). Blood gas parameters (pH, pCO_2_, HCO3, and lactate) at admission were not different. The need for intubation within 72 h as the primary outcome was similar between NCPAP and NIPPV groups (35.5 vs. 34.5%, *p* = 0.935). The rates of surfactant requirement, need for intubation within 7 days, prematurity-related morbidities, mortality, and death/BPD were similar among the groups (*p* > 0.05).

**Conclusion:**

Nasal intermittent positive pressure ventilation is non-inferior to NCPAP as an initial treatment in extremely preterm infants with RDS. Although the rate of intubation in the first week, mortality, and BPD did not differ between groups, additional studies are needed and the synchronization of NIPPV should be evaluated.

## Introduction

The most common respiratory morbidity in preterm infants is respiratory distress syndrome (RDS). Infants with RDS frequently require any level of respiratory support. Invasive ventilation strategy and prolonged tracheal intubation are associated with bronchopulmonary dysplasia (BPD). Therefore, it is common to use non-invasive respiratory support in the treatment of RDS to reduce lung injury and BPD (1).

Non-invasive ventilation is preferred to use instead of invasive mechanical ventilation (1–3). Nasal continuous positive airway pressure (NCPAP) and nasal intermittent positive pressure ventilation (NIPPV) are the most commonly used non-invasive respiratory support modalities in RDS compared with others as high flow nasal cannula, nasal high-frequency oscillatory ventilation, neurally adjusted ventilator assist. NCPAP reduces upper airway resistance, helps to establish functional residual capacity, stabilizes the chest wall, and preserves endogenous surfactant (4). NIPPV was also studied as another method for the RDS treatment. It is a time-cycled pressure-limited ventilation mode, superimposes an intermittent peak pressure on NCPAP. In addition to the benefits of NCPAP, NIPPV improves ventilation in apnea by providing a backup rate and using sufficient peak inspiratory pressure. Due to the use of higher mean airway pressure, NIPPV results in better alveolar recruitment and improved carbon dioxide clearance (4–6).

Following the clinical trials, the systematic review of the Cochrane on NIPPV for initial support of neonatal RDS reported NIPPV to be superior to NCPAP for decreasing respiratory failure and the need for intubation and endotracheal tube ventilation (7), however, evidence is limited.

In this study, we aimed to compare NCPAP vs. NIPPV as an initial treatment of RDS in terms of prematurity-related morbidities and mortality.

## Materials and Methods

### Study Population

Data of infants who were born before 29 weeks' gestation and admitted to the Ankara University Children's Hospital Neonatal Intensive Care Unit (NICU) between 1 January 2018 and 31 December 2021 were evaluated for eligibility. A simple random sampling method was used. Preterm infants were excluded who were outborn had congenital malformations (any airway abnormalities, major cardiac malformations, and disorders requiring surgery), and were intubated in the delivery room or the first 6 h after birth. Infants who received non-invasive respiratory support with the first episode of NCPAP or NIPPV as initial treatment were included. Infants were assigned to the NCPAP and NIPPV groups randomly.

The study protocol was approved by the Ankara University Faculty of Medicine Ethics Committee. Consent was waived by the ethics board in the light of retrospective nature of the study design.

### Study Design

Data were extracted for baseline demographics as gestational age (GA), birth weight (BW), gender, antenatal glucocorticoid administration, maternal chorioamnionitis, delivery room resuscitation, APGAR scores at 5^th^ min, and blood gas parameters (pH, pCO_2_, HCO_3_, and lactate) at admission.

Nasal continuous positive airway pressure is used in the delivery room for all the infants born at and before 32 weeks' gestation who have spontaneous breathing as recommended by the RDS guideline of the Turkish Neonatal Society. T-piece resuscitators with short bi-nasal prongs are used to provide prophylactic NCPAP at a pressure of at least 5 cmH_2_O. During transport to the NICU, NCPAP was applied with T-piece resuscitators integrated into the transport incubator (3).

Infants who do not breathe within the first 60 s after birth or who remain bradycardic (heart rate <100 per min) despite appropriate first interventions (including tactile stimulation) were administered positive pressure ventilation (PPV) at a rate of 40–60 per min (min). If the heart rate is <60 beats per min after at least 30 s of adequate PPV, chest compressions have been initiated. Before starting chest compressions, ventilation has been optimized, preferably with endotracheal intubation. PPV and all subsequent steps are referred to as delivery room resuscitation (8). Since infants intubated in the delivery room were excluded from the study, the presence of delivery room resuscitation only refers to PPV.

Ventilator settings were at the discretion of the medical team and adjusted to target pulse oxygen saturation of 90–95% with a rate of 20–40 breaths per min, peak inspiratory pressure of 15–20 cmH_2_O, peak end expiratory pressure of 5–7 cmH_2_O in NIPPV group which was provided by Sophie (Stephan, Germany) ventilators *via* nasal mask and/or bi-nasal prongs. In the NCPAP group, the PEEP range was between 5 and 7 cmH_2_O which was provided by Sophie (Stephan, Germany) or Infant Flow Driver (Viasys, Carefusion, USA).

Surfactant was indicated when PEEP was >7 cmH_2_O and FiO_2_ was >40% according to the Turkish Neonatal Society guideline on the management of respiratory distress syndrome and surfactant treatment (3). Infants received surfactant *via* less invasive surfactant administration (LISA) or INSuRE (INtubate, SURfactant, and immediate Extubation) technique if they needed.

### Primary and Secondary Outcomes

The need for intubation within 72 h was the primary outcome. The following were evaluated as secondary outcomes: The need for intubation in 7 days, rates of surfactant administration, early-onset sepsis (EOS), hemodynamically significant patent ductus arteriosus (hsPDA) (9), intraventricular hemorrhage (IVH) (≥ grade 3) (10), late-onset sepsis (LOS) (proven or clinical), necrotizing enterocolitis (NEC) (≥stage 2) according to the Bell staging (11), retinopathy of prematurity (ROP) according to the International Classification of Retinopathy of Prematurity (12), BPD (moderate or severe) according to National Institutes of Health (NIH) criteria (13), and mortality.

### Statistical Analysis

All the statistical analyses were performed using SPSS for Windows version 11.5 software (SPSS Inc., Chicago, IL, US). Descriptive statistics were expressed as means ± SDs and median (minimum–maximum) for quantitative variables and the number (percent) for qualitative variables. The compatibility of data with normal distribution was examined graphically and using the Kolmogorov–Smirnov test. When to look whether there was a statistically significant difference between the categories of a qualitative variable with two categories in terms of a quantitative variable, Student's *t*-test was used if the normal distribution assumption was met; if not, the Mann–Whitney *U* test was used. The chi-square test and Fisher-exact test were used to examine the relationship between two categorical variables. Logistic regression was used for analyzing of independent variables that determine the risk factors for a dependent qualitative variable with two categories, which was a statistically significant risk factor for this variable. A *p*-value of 0.05 was considered statistically significant.

## Result

**Figure 1:**
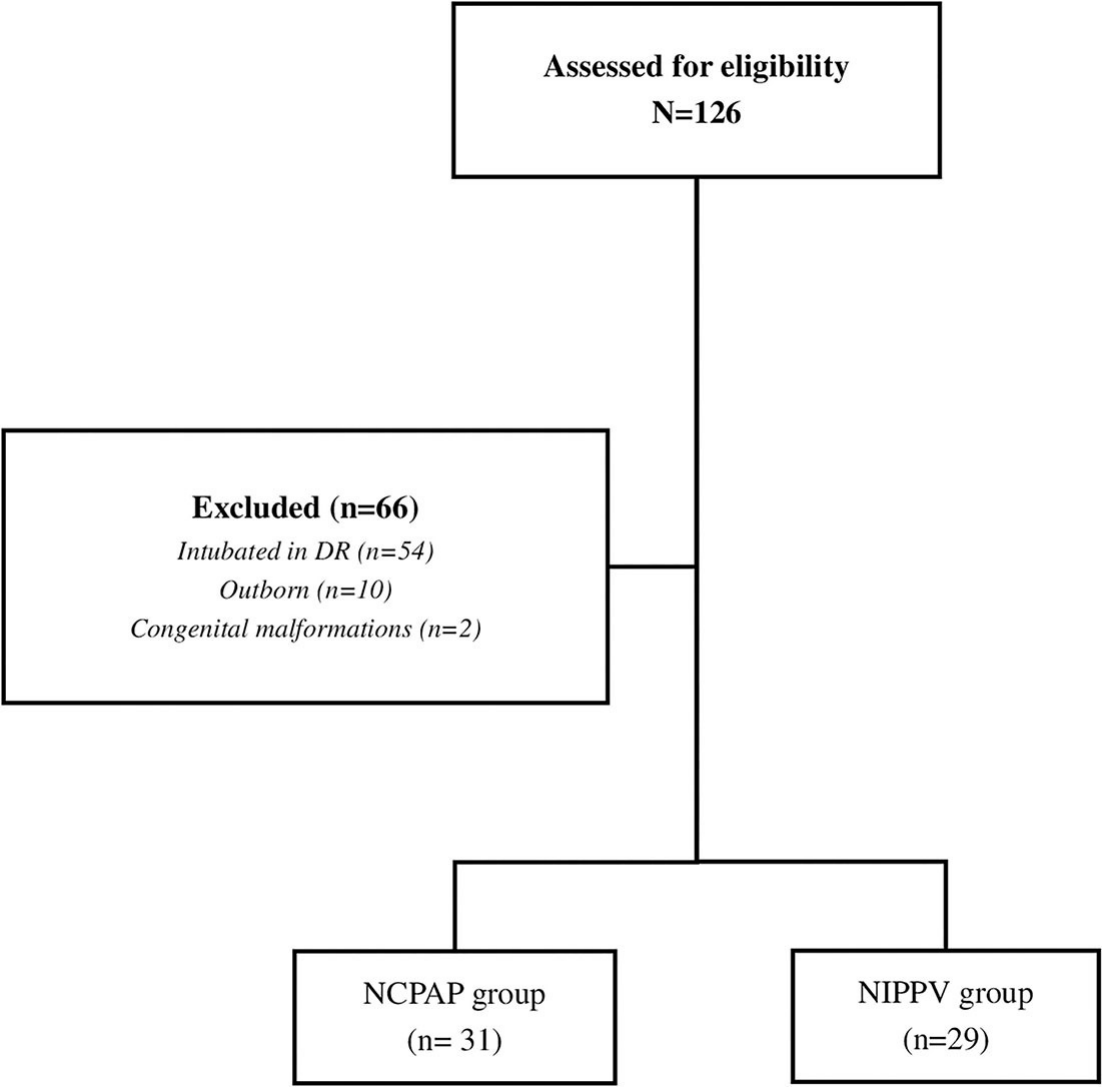
Flowchart of the participants.

During the study period, there were 116 inborn admissions of preterm neonates born <29 weeks' gestation to our NICU. A total of 60 patients were included according to inclusion criteria. Of these, 31 (52%) infants received NCPAP, while 29 (48%) received NIPPV in the first several hours after birth (Figure 1). In the first 24 h of life, no patient received solely oxygen without pressure requirement. The demographic and clinical findings regarding GA, BW, gender, administration of antenatal glucocorticoids, maternal chorioamnionitis, delivery room resuscitation, Apgar score at 5^th^ min, and rate of EOS were similar between the groups (*p* > 0.05) (Table 1).

**Table 1:** Demographic and clinical conditions of the groups.

	**NCPAP (*n* = 31)**	**NIPPV (*n* = 29)**	***p*-value**
Gestational age (w)^*^^,^ ^†^	26.49 ± 1.38	26.37 ± 1.38	0.735^a^
	26.50 (24.00–28.60)	26.30 (23.50–28.60)	
Birth weight (g)^*^^,^ ^†^	947 ± 264	860 ± 211	0.167^a^
	855 (445–1,650)	850 (460–1,460)	
Male sex, *n* (%)	21 (67.7)	15 (51.7)	0.206^c^
Multiple birth, *n* (%)	8 (25.8)	9 (31)	0.653^c^
Antenatal glucocorticoids, *n* (%)	23 (74.2)	21 (72.4)	0.876^c^
Maternal chorioamnionitis, *n* (%)	7 (22.6)	10 (34.5)	0.307^c^
Delivery room resuscitation, *n* (%)	7 (22.6)	3 (10.3)	0.302^d^
Apgar score at 5^th^ min^*^^,^ ^†^	7.81 ± 1.05	7.31 ± 0.97	0.074^b^
	8.00 (5.00–10.00)	7.00 (4.00–9.00)	
Early-onset sepsis, *n* (%)	21 (67.7)	19 (65.5)	0.855^c^
Hospitalization duration (d)^*^^,^ ^†^	45.52 ± 38.23	52.28 ± 38.32	0.332^b^
	42.00 (0.00–182.00)	61.00 (0.00–114.00)	

*a: Student t-test, b: Mann Whitney U test, c: Chi-square test d: Fisher-exact test*.

* *Plus–minus values are means ± SD*;

† *Values in parenthesis are medians (min–max)*.

*NCPAP, nasal continuous positive airway pressure; NIPPV, nasal intermittent positive pressure ventilation*.

Table 2 lists the initial blood gas parameters of infants at admission. There were no significant differences between pH, PCO_2_, HCO_3_, and lactate between NCPAP and NIPPV groups (*p* > 0.05).

**Table 2:** Initial blood gas parameters at admission.

**Variables**	**NCPAP (*****n*** **=** **31)**		**NIPPV (*****n*** **=** **29)**		
	**Mean ±SD**	**Median (Min-Max)**	**Mean ±SD**	**Median (Min-Max)**	***p*-value**
pH	7.28 ± 0.06	7.27 (7.20–7.39)	7.26 ± 0.06	7.24 (7.19–7.41)	0.565^b^
PCO_2_	45.03 ± 9.08	44.90 (31.30–63.70)	47.24 ± 8.59	47.10 (25.00–60.80)	0.436^a^
Lactate	2.53 ± 1.34	2.10 (1.00–5.60)	2.58 ± 1.91	2.20 (0.80–10.40)	0.876^b^
HCO_3_	20.44 ± 3.05	21.60 (14.50–24.50)	20.54 ± 3.14	20.50 (12.70–25.20)	0.927^a^

*a: Student t-test, b:Mann–Whitney U test*.

*HCO_3_, bicarbonate; NCPAP, nasal continuous positive airway pressure; NIPPV, nasal intermittent positive pressure ventilation; PCO2, partial pressure of carbondioxide; pH, power of hydrogen; SD, standard deviation*.

The rate of intubation within 72 h was 35.5% of the infants in the NCPAP group and 34.5% in the NIPPV group which was similar (*p* = 0.935). The rate of intubation within 7 days was also similar between the groups (45.2 vs. 51.7%, *p* = 0.611). In total, fourteen infants (45.2%) in the NCPAP group and 16 infants (55.2%) in the NIPPV group received surfactant which was not statistically significant (*p* = 0.438). There were no differences regarding prematurity-related morbidities between the groups (Table 3).

**Table 3:** Primary and secondary outcomes.

**Variables**	**NCPAP (*n* = 31)**	**NIPPV (*n* = 29)**	***p*-value**
Intubation within 72 h	11 (35.5)	10 (34.5)	0.935^a^
Intubation within 7 d	14 (45.2)	15 (51.7)	0.611^a^
Surfactant requirement	14 (45.2)	16 (55.2)	0.438^a^
LOS	20 (66.7)	23 (82.1)	0.179^a^
hsPDA	13 (43.3)	13(46.4)	0.813^a^
IVH (≥ Grade 3)	4 (13.3)	4 (14.3)	1.000^b^
NEC (≥ Stage 2)	7 (23.3)	7 (25.0)	0.882^a^
ROP (≥ Stage 3)	4 (12.9)	2 (6.9)	0.672^a^
Death within 3 days	3 (9.7)	1 (3.4)	0.613^b^
Death within 7 days	4 (12.4)	4 (13.8)	1.000^b^
Death	10 (32.3)	7 (24.1)	0.485^a^
Moderate or severe BPD	10 (33.3)	16 (57.1)	0.068^a^
Death/BPD (Moderate or severe)	20 (64.5)	23 (79.3)	0.204^a^
Severe BPD	3 (9.7)	4 (13.8)	0.702^b^
Death/BPD (Severe)	13 (41.9)	11 (37.9)	0.752^a^

*Data was given as n (%)*.

*a: Chi-square test, b: Fisher-exact test*.

*BPD, bronchopulmonary dysplasia; hsPDA, hemodynamically significant patent ductus arteriosus; IVH, intraventricular hemorrhage; LOS, late onset sepsis; NCPAP, nasal continuous positive airway pressure; NEC, necrotizing enterocolitis; NIPPV, nasal intermittent positive pressure ventilation, ROP, retinopathy of prematurity*.

The incidence of BPD (moderate or severe) was similar between the groups, with a slighter prevalence in the NCPAP group (33.3 vs. 57.1%). Only 3 (9.7%) infants in the NCPAP group and 4 (13.8%) infants in the NIPPV group developed severe BPD (OR: 1.493, 95% CI, 0.304–7.331, *p* = 0.621).

The rate of mortality was similar between two groups (32.3 vs. 24.1%; OR: 0.668, 95% CI, 0.215–2.081, *p* = 0.487). In total, four (12.4%) infants in the NCPAP group and 4 (13.8 %) infants in the NIPPV group had died before the postnatal 7^th^ day. All the deaths occurred before 36 weeks of postmenstrual age. The composite rate of severe BPD or death for the NCPAP group was 41.9%, whereas it was 37.9% for the NIPPV group (OR: 0.846, 95% CI, 0.301–2.382, *p* = 0.752) (Table 4).

**Table 4:** The effect of using NIPPV instead of NCPAP.

**Variables (reference)**	**β**	**S.E**.	***p*-value**	**Odds ratio**	**95% C.I. for Exp (β)**	
					**Lower**	**Upper**
Intubation within 72 h	−0.044	0.542	0.935	0.957	0.331	2.767
Intubation within 7 d	0.263	0.518	0.611	1.301	0.471	3.591
Death within 3 days	−1.099	1.185	0.354	0.333	0.033	3.402
Death within 7 days	0.077	0.760	0.919	1.080	0.244	4.787
LOS	0.833	0.627	0.184	2.300	0.673	7.864
PDA (hsPDA)	0.125	0.529	0.813	1.133	0.402	3.193
IVH (≥ Grade 3)	0.080	0.762	0.916	1.083	0.243	4.820
NEC (≥ Stage 2)	0.091	0.614	0.882	1.095	0.329	3.648
ROP (≥ Stage 3)	−0.693	0.908	0.445	0.500	0.338	11.851
Death	−0.403	0.580	0.487	0.668	0.215	2.081
Moderate and severe BPD	0.981	0.544	0.071	2.667	0.918	7.744
Death/BPD (moderate or severe)	0.746	0.592	0.208	2.108	0.660	6.734
Severe BPD	0.401	0.812	0.621	1.493	0.304	7.331
Severe BPD or death	−0.167	0.528	0.752	0.846	0.301	2.382

*SE, Standard error of mean, CI, Confidence Interval*.

*BPD, bronchopulmonary dysplasia; hsPDA, hemodynamically significant patent ductus arteriosus; IVH, intraventricular hemorrhage; LOS, late onset sepsis; NEC, necrotizing enterocolitis; ROP, retinopathy of prematurity*.

## Discussion

This single-center study compared the two non-invasive respiratory modalities as an initial treatment for RDS in extremely preterm infants to avoid intubation within 72 h after birth. Based on the findings, no significant difference was found with using either NIPPV or NCPAP for intubation requirement within both 72 h and the first week of life besides prematurity morbidities except for BPD. The rate of mortality was higher in infants who received NCPAP group, whereas the incidence of BPD was higher in the NIPPV group. Since the goal of this study was to investigate the effects of different non-invasive respiratory support modalities in the first several hours of life, the transition after the first day between non-invasive and invasive respiratory support modalities has not been evaluated.

Despite the increasing use of NIPPV as an initial respiratory support for extremely preterm infants, the literature remains lack of strong evidence. A recent Cochrane review comparing NIPPV vs. NCPAP use as a primary mode in preterm infants showed a benefit with the use of NIPPV with less respiratory failure and need for intubation. The most important disadvantage of these studies is the heterogeneity of the gestational weeks of the infants included. The authors had planned subgroup analyses based on GA (28 vs. >28 weeks) and BW (1,000 g vs. >1,000 g), but they were unable to demonstrate any advantage on the other hand no overall reduction in the risk of chronic lung disease (CLD) (7). In this study, no superiority was observed between the two modalities in terms of intubation requirement or BPD occurrence.

The study by Kirpalani et al., which is the most important study that directs the meta-analysis, found no significant benefit of NIPPV with respect to the risk of BPD or death (14). In the sub-study from the same cohort, 27.5% of infants in the NIPPV group and 30.1% of infants in the NCPAP group failed non-invasive support within the first 7 days of life with a relative risk of 0.91. (95% CI, 0.54–1.53) (15). Li et al. conducted another meta-analysis to evaluate whether NIPPV would decrease the intubation need compared with NCPAP for preterm infants with RDS, and found that NIPPV could not decrease the need for invasive ventilation (16). In this study, no evidence was found in increasing the risk of intubation in the first week of life within infants received either NCPAP or NIPPV.

The failure of non-invasive respiratory support in the NCPAP and nasal intermittent mandatory ventilation (NIMV) groups were evaluated in research by Armanian et al. in infants whose birth weight was ≤ 1,500 g and/or gestational age were ≤ 34 weeks. In each group, the requirement for the mechanical ventilation in the first 48 h of life, and also the duration of non-invasive respiratory support was investigated. They reported that NCPAP and NIMV had the same effects in preventing invasive mechanical ventilation in the first 48 h of life similar to this study. But, the duration of non-invasive ventilation and oxygen dependence, and length of hospital stay were also shorter in the NIMV group (17). Dursun et al. compared NCPAP and NIPPV in a prospective study including infants younger than 32 weeks' gestation. The aim of the study was to evaluate the effects of these modalities in terms of invasive mechanical ventilation requirements. Endotracheal intubation rates were found to be significantly lower in the NIPPV group than in the NCPAP group. BPD and the other early morbidities were not different between the groups (18). It is important to point out that those two studies included infants in a wider range of GA and BW who are larger preterms which might have caused to reveal differences between NCPAP and NIPPV groups. Different results could have been obtained with other researches since this study included a more homogeneous patient population and extremely preterm infants.

This study by Ahmad et al. comparing NCPAP (with a high pressure of 9 cmH_2_O) and NIPPV found no difference regarding intubation within 7 days. There was no difference in air leakage, NEC, or feeding intolerance between the groups as secondary outcomes (19). Their study population was similar to the present one (<29 weeks' gestation), however, no difference has not been demonstrated at higher CPAP pressures in these extremely preterm patients.

In the previous studies that compared NCPAP to NIPPV, researchers used a ventilator or a bi-level device to deliver NIPPV. This might lead to an incorrect appraisal of the NIPPV ventilator's effectiveness. In this study, two different ventilators were used for CPAP support, however, all the patients in the NIPPV group were ventilated by only one type of device. Unlike several prior studies, no patient received bi-level positive airway pressure with IFD (infant flow driver). In a recently published meta-analysis, trials were compared based on device and synchronization. The authors added 850 data of infants from 8 newly published trials to the existing Cochrane meta-analysis (20). They found that the beneficial effect of NIPPV was most obvious in the ventilator-generated synchronize NIPPV. The mortality rate was similar between NCPAP and NIPPV groups. Because the NIPPV group did not differ in terms of synchronization and non-synchronization in our study population, we may not have detected a reduction in BPD risk in the NIPPV group.

The retrospective nature and small sample size are the main limitations of this study. It might have been caused by a bias in the clinical decision of the medical team on the preference of NCPAP vs. NIPPV. In this study, the evidence is limited to suggest one mode to another; however, the use of NIPPV as a second-line treatment at NCPAP failure in our unit in previous years may have led the attending neonatologist to prefer NIPPV in clinically ill infants. The homogeneity of the study as GA and BW is one of the strengths of this study, and also standardized ventilators were used during the study period.

In a conclusion, NIPPV is non-inferior from NCPAP with respect to short-term respiratory outcomes in this small sample-sized cohort. No other clinically important outcomes differed significantly between the groups. Future randomized controlled studies and strong evidences will be needed.

## Data Availability Statement

The raw data supporting the conclusions of this article will be made available by the authors, without undue reservation.

## Ethics Statement

The studies involving human participants were reviewed and approved by Ankara University Faculty of Medicine Local Ethics Committee. Written informed consent from the participants' legal guardian/next of kin was not required to participate in this study in accordance with the national legislation and the institutional requirements.

## Author Contributions

YEK, EO, and SA gave a substantial contribution in article conception and design. YEK, OE, and BA participated in the acquisition of data. BB performed statistical analysis of the data. YEK and EO drafted the manuscript. SA critically revised it. All the authors gave their final approval to this manuscript and agree to be accountable for all aspects of the work ensuring integrity and accuracy.

## Conflict of Interest

The authors declare that the research was conducted in the absence of any commercial or financial relationships that could be construed as a potential conflict of interest.

## Publisher's Note

All claims expressed in this article are solely those of the authors and do not necessarily represent those of their affiliated organizations, or those of the publisher, the editors and the reviewers. Any product that may be evaluated in this article, or claim that may be made by its manufacturer, is not guaranteed or endorsed by the publisher.
